# Concentration of soil-transmitted helminth eggs in sludge from South Africa and Senegal: A probabilistic estimation of infection risks associated with agricultural application

**DOI:** 10.1016/j.jenvman.2017.12.003

**Published:** 2018-01-15

**Authors:** Isaac Dennis Amoah, Poovendhree Reddy, Razak Seidu, Thor Axel Stenström

**Affiliations:** a SARChI Chair, Institute for Water and Wastewater Technology, Durban University of Technology, PO Box 1334, Durban, 4000, South Africa; b Department of Community Health Studies, Faculty of Health Sciences, Durban University of Technology, PO Box 1334, Durban, 4000, South Africa; c Water and Environmental Engineering Group, Institute for Marine Operations and Civil Engineering, Norwegian University of Science and Technology, Ålesund, Norway

**Keywords:** Sludge reuse, South Africa, Senegal, QMRA, Soil-transmitted helminths, *Ascaris*

## Abstract

The use of sludge in agriculture has been encouraged as a means of increasing soil nutrient content and improving the water holding capacity. On the negative side, major public health concerns with sludge application prevail, mainly due to the high concentration of pathogenic microorganisms. Soil-transmitted helminths (STHs) are of major health concern in this regard, especially in endemic regions, mainly due to the high environmental resistant of the eggs combined with a low infectious dose. In this study the concentration of STH eggs in two months dried sludge from Durban, South Africa and Dakar, Senegal was determined and compared. Sampling was carried out from January to October 2016 and in September 2016 for Dakar. *Ascaris* spp, hookworm, *Trichuris* spp, *Taenia* spp and *Toxocara* spp were the commonly recorded STH eggs. STH egg concentrations were higher in Dakar than in Durban, with viable STH egg concentrations exceeding both local and international guidelines. Due to the high concentration of viable STH eggs, risks of *Ascaris* spp infection was very high for farmers applying this sludge on their farms in both Durban (7.9 × 10^−1^ (±1.7 × 10^−2^)) and Dakar (9.9 × 10^−1^ (±1.3 × 10^−5^)). Consumption of lettuce grown on sludge amended soil will result in probable infections but harvest after 30 days between sludge application and harvest in Durban gave median probability infection risks with a risk level similar to the WHO tolerable risk value (10^−4^). This time period need to be prolonged to harvest in Dakar to 40 days to reduce the risks of infection to the tolerable risks values. Further treatment of the sludge either through composting or drying for longer periods of time is thus recommended from a public health perspective.

## Highlights

• Sludge contained high concentration of viable STH eggs after 60 days of drying.
• The concentration of these eggs exceeded both national and international guidelines for reuse.
• Farmers and consumers of the farm produce are likely to be infected with *Ascaris* spp.
• Further treatment of the sludge is thus recommended from a public health perspective.

## 1 Introduction

Application of faecal sludge on agricultural lands is a major component of resource recovery, aimed to ensure that vital plant nutrients are returned to the soil. Sludge is high in organic matter and nutrients (Hernández et al., 2017), making it a valuable amendment in restoring degraded, exhausted (Jiménez and Álvarez, 2005) and burned soils (Guerrero et al., 2007), as well as improving fertility and crop productivity (Bittencourt et al., 2013). It may also increase the water holding capacity of soil (Navarro-Pedreño et al., 2003, Almendro-Candel et al., 2014).

Despite the recognition of faecal sludge application globally, there are public health concerns regarding its quality and impact on human health. The physico-chemical and biological processes involved in wastewater and sludge treatment will concentrate several harmful substances, such as metals/metalloids (Castells, 2012) and most importantly pathogenic bacteria (Krzyzanowski et al., 2016, Pepper et al., 2008, Jiménez et al., 2007) and helminths (Gerba and Smith, 2005, Navarro et al., 2009, Murcia Navarro, 2013).

The most important microbial health risk when applying sludge to agricultural soils are the soil-transmitted helminths (STHs) (WHO, 2006). The presence of these parasite eggs is considered as an indication of the health hazard of sludge after application, due to the very high resistance of the eggs (Zdybel et al., 2015: Gaspard et al., 1995) to adverse environment conditions (Melvin et al., 2001, Nelson and Darby, 2001), resulting in long-term environmental contamination (Johnson et al., 1998). In addition, the occurrence of these eggs correspond to a very low infectious dose (Toze, 2006, Crompton and Nesheim, 2002, Stephenson et al., 2000). Poor sanitation and faecal contamination result in an enhanced transmission and a high prevalence in developing countries, where faecal sludge is a main contributing factor (67–735 eggs/g total solids (TS)) (Feachem et al., 1983, Strauss et al., 2003, Jiménez and Wang, 2006) as compared to more developed countries (2–13 eggs/g TS) (Jiménez et al., 2002).

To protect humans from the health impact of STH infections as a result of sludge application in agriculture, many countries have set up national regulations to treat and reuse sludge (Navarro et al., 2009). These regulations are adopted from the limits for STH eggs stated by the United States Environmental Protection Agency (USEPA) (1993) and expressed as a guideline value by World Health Organization (WHO) (2006), namely 0.25 and 1 helminth egg/g TS respectively. Countries such as Brazil, Chile, New Zealand and South Africa adopted the regulations based on the USEPA limits (Jiménez et al., 2002) for Class A sludge that is intended for use in unrestricted agriculture, but monitoring is very rarely done. To meet these international and national guidelines, sludge undergoes further treatments, such as air-drying, composting or long-term storage (O'Connor et al., 2017). However long-term storage (>3 years) results in loss of key plant nutrients (Grant et al., 2012). Reduction in viable STH egg concentrations during air-drying is dependent on ambient temperature and duration of drying in addition to several environmental factors, such as irradiation, rainfall etc. (Grant et al., 2012).

The regulations proposed by the USEPA and the WHO were based on sparse epidemiological data rather than risk assessment estimates (Navarro et al., 2009). The use of the quantitative microbial risk assessment (QMRA) approach to establish the health risks involved in the application of sludge in agriculture was done after the development of these guidelines and have been applied in Australia (NWQMS, 2006). QMRA is a probabilistic approach to predict human health risks from exposure to pathogenic microorganisms and it has been used extensively in assessing the transmission of water (Smeets et al., 2010, McBride et al., 2013, Bichai and Smeets, 2013) and food borne (Janevska et al., 2010, Romero-Barrios et al., 2013, Membré and Boué, 2017) infections. QMRA has also been used in estimating STH infection risks from the reuse of wastewater and sludge in agriculture (Jiménez et al., 2002, Navarro and Jiménez, 2011, Navarro et al., 2008, Kundu et al., 2014, Seidu et al., 2008a, Seidu et al., 2008b).

This study contributes to the knowledge on STH egg concentration in sludge from different populations by comparing the total and viable STH egg concentrations in sludge from two large scale wastewater treatment plants in Durban, South Africa and one plant in Dakar, Senegal. The suitability of sludge, after approximately 60 days of drying (without mixing) under ambient environmental conditions, for agricultural application was determined using the QMRA approach. This study therefore contributes to the evidence necessary for the revision of sludge application guidelines depending on the type of treatment.

## 2 Methodology

### 2.1 Study area and sampling

Sludge samples were taken from two centralized wastewater treatment plants (WWTPs) in Durban, South Africa. One has an operational capacity of 10.98 Ml/d (WWTP A) and the second smaller one with a capacity of 4.69 Ml/d (WWTP B). Each of these plants have sludge drying beds were sludge is dried for a duration of 60–90 days (depending on demand and availability of drying beds) before disposal. Sludge sampling in Senegal was from a WWTP treating wastewater in the city of Dakar, which has an operational capacity of about 13 Ml/d. Composite samples were taken in triplicates from the drying beds from January to October 2016 for the two WWTPs in Durban and in September 2016 for the plant in Dakar.

The decay of viable STH eggs during drying over a 90 day period was also determined by sampling from an experimental bed at WWTP A, starting from the first day of drying to 90 days. Composite samples were taken in triplicates from different depths of the same sludge bed, by dividing the depth into three, top, middle and bottom layers.

### 2.2 Laboratory analysis

Sludge analysis for STH eggs was performed using a revised method based on the principles of flotation and sedimentation, as compared in Amoah et al. (2017). Briefly, samples were homogenized and approximately 20 g sludge portions measured into 250 mL to which 50 mL of ammonium bicarbonate (119 g/L) (Sigma Aldrich, Germany) was added and allowed to soak for 10 min. The samples were then blended at top speed, with a laboratory blender (Isolab Laborgeräte GmbH) and poured through a 100 μm sieve onto a 20 μm sieve. The contents on the 100 μm sieve were washed carefully with tap water under pressure, to separate all STH eggs from particulate matter and enable the eggs pass through the pores. The contents collected on the 20 μm sieve were then carefully washed and collected into a 50 mL centrifuge tube and centrifuged for 10 min at 3000 rpm. The supernatants were discarded and ${\text{ZnSO}}_{4}$ (Promark Chemicals, South Africa) solution of specific gravity 1.30 added to the pellets. The pellets were then re-suspended in the ${\text{ZnSO}}_{4}$ by vortexing and centrifuged again at 2000 rpm for another 10 min. The supernatants was poured through the 20 μm sieve and washed carefully with tap water, to remove all residual ${\text{ZnSO}}_{4}$, and the contents collected into 50 mL centrifuge tubes and centrifuged at 3000 rpm for 10 min. The supernatants were finally discarded and pellets viewed under ×10 magnification (Leica DM2000) to determine the total STH eggs per gram of the sludge analyzed. STH eggs on the positive slides were carefully washed into a petri dish for incubation at room temperature with 0.1N sulphuric acid (Promark Chemicals, South Africa) as an incubation solution for 28 days, after which the viable STH eggs were counted by viewing under ×40 magnification. STH eggs with a visible motile larva were considered as viable.

### 2.3 Statistical analysis

Descriptive analysis of the STH egg concentrations and distribution was performed using Excel (Microsoft Corporation). Difference in concentration of the various types of STH eggs was determined using the Kruskal-Wallis tests, with the Mann-Whitney *U* test used to compare the difference in concentrations between the total and viable STH eggs as well as difference in egg concentrations between the sampling sites. All statistical analysis was performed in Graphpad Prism 7 software (GraphPad Software, Inc. USA).

### 2.4 Assessment of STH infection risks

Risk of infection with STH was determined using the QMRA approach, which involves four interrelated steps: a) hazard identification; b) exposure assessment; c) dose-response assessment and d) risk characterization (Haas et al., 2014).

#### 2.4.1 Hazard identification

For the purpose of this assessment *Ascaris* spp was chosen as an index for STHs. The link between ascariasis and wastewater/sludge reuse in agriculture has been established by several reports (Ensink et al., 2008, Fuhrimann et al., 2014, Fuhrimann et al., 2016, Amoah et al., 2016, Contreras et al., 2017), with risks successfully estimated using the QMRA approach (Seidu et al., 2008a, Seidu et al., 2008b, Navarro et al., 2009). Additionally *Ascaris* spp is the only STH with a dose-response model.

#### 2.4.2 Exposure assessment

This step involves the determination of number of *Ascaris* spp eggs that will be ingested per single exposure. Accidental ingestion of the sludge during the application was considered the first point of exposure. Westrell et al. (2004) used an ingestion weight of 2 g, but in this study we assumed that the weight of sludge ingested will be uniformly distributed from 1-2 g. Using lettuce as a surrogate for vegetables grown on sludge amended soils, it was further assumed that the sludge will be spread before each growing season which translates to 7–12 times per year, considering that most lettuce varieties take 30–50 days to mature (CTAHR, 2000).

Ingestion of soil amended with sludge was also considered, where it was expected that there will be a dilution of the sludge in the soil in addition to decay of the *Ascaris* spp eggs over time. Therefore the concentration of STH eggs in the sludge amended soil on the day of application was calculated using the formula;

(1) $$N_{o}=C_{sludge}\times Df$$

where “*N*_*o*_” is the concentration of STH per gram of soil after spreading on the day of application, “*C*_*sludge*”_ the concentration of STH eggs in the sludge, as determined in this study and “*Df*” the dilution factor, which was taken to be 0.001, assuming a sludge to soil ratio of 1:100 (Schönning et al., 2007). Taking into consideration the decay of the *Ascaris* spp eggs over time, the concentration ingested days after application was determined using the formula:

(2) $$N_{t}=No*e^{-kt}$$

where *N*_*t*__is_ the concentration of viable *Ascaris* spp eggs at time *t* (measured in days), *N*_*o*_ is the concentration of viable eggs on the day of sludge application as determined in equation 1 and *k* is the decay constant of *Ascaris* spp eggs, which was calculated to be 0.0056 based on results obtained during the egg decay analysis (See Table A1 in Appendix for Data).

It was assumed that farmers will ingest 0.03–0.1 g of soil per day (Schönning et al., 2007). Additionally, frequency of ingestion was assumed to be uniformly distributed from 64 to 128 days in a year to account for the labour-intensive nature of agriculture in developing countries.

We also hypothesized that consumption of the lettuce may result in *Ascaris* spp infections. Westrell et al. (2004) used an assumption of 1 g of sludge per each serving of raw vegetables grown on sludge amended soils but in this study we assumed that the amount of sludge ingested through this route would be uniformly distributed from 0.5 g to 1 g per serving with an annual frequency of 156–160 per year. This risk was however calculated for 30–50 days after application of the sludge, to account for time needed for lettuce to mature. Table 1 below presents the various assumptions made in the estimation of risk based on the exposure scenarios.

**Table 1:** Points of exposure with assumptions based on weights of sludge ingested and frequency of exposure.

Exposure scenario	Weight of sludge ingested (g) per day	Frequency	Reference
Spreading on sludge	Uniform distribution (1,2)	Uniform distribution (7,12)^a^	Westrell et al., 2004 (Maximum weight of 2 g per event)
Ingestion of sludge amended soil	Uniform distribution (0.03,0.1)	Uniform distribution (64,128)^a^	Schönning et al., 2007
Ingestion of sludge through raw vegetables	Uniform distribution (0.5,1)	Uniform distribution (156,160)^a^	Westrell et al., 2004 (Maximum weight of 1g per day)

a Assumptions made in this study.

#### 2.4.3 Dose-response assessment

The exponential dose-response model as proposed by Navarro et al. (2008) was used. This model has been used in estimation of *Ascaris* spp infection risks by other researchers (Westrell et al., 2004, Seidu et al., 2008a). Therefore the risk of *Ascaris* spp infection was assessed using the formula

(3) $$P_{inf}=1-e^{-rd}$$

where “*P*_*inf*_*”* is the infection risk per exposure event, “*d”* the number of *Ascaris* spp eggs ingested per that event and “*r”* the dimensionless infectivity constant. An “*r”* value of 0.039 as reported by Navarro et al. (2008) was used in this study.

#### 2.4.4 Risk characterization

All the outcomes of the hazard identification, exposure assessment and dose response assessment were combined to determine the severity of *Ascaris* spp infection from the different exposure scenarios considered. Risk of infection due to multiple exposures or annual risk (*P*_*A*_) was determined using the formula:

(4) $$P_{A}=1-{\left(1-P_{inf}\right)}^{n}$$

$P_{inf}$ is the risk of infection from a single exposure event and *n* being the frequency of exposure per year (Sakaji and Funamizu, 1998). All models used for the risk of infection determination was constructed in Microsoft Excel using @Risk 7.5 (Palisade Corporation) software add-on to Excel and subjected to Monte-Carlo simulations of 10, 000 iterations.

## 3 Results

### 3.1 Concentration of STH eggs in sludge from Durban, South Africa

**Fig. 1:**
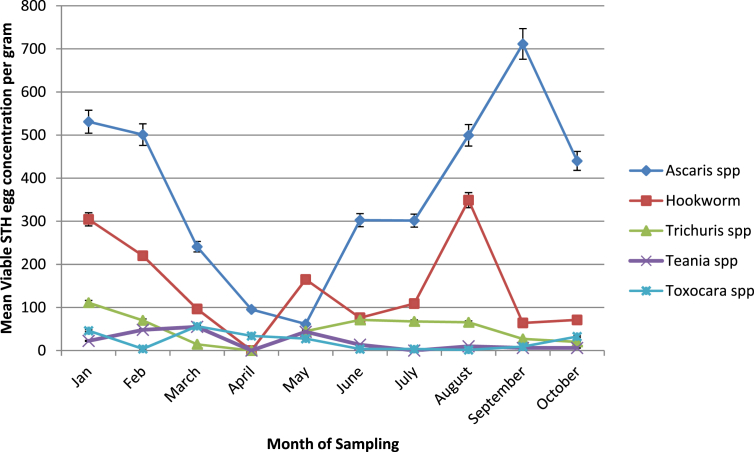
Variation in viable STH egg concentrations during the study in Durban, South Africa.

*Ascaris* spp, hookworm, *Trichuris* spp, *Taenia* spp and *Toxocara* spp were detected in sludge from the two wastewater treatment plants (WWTPs) in Durban. The difference in egg concentrations between these two WWTPs was not statistically significant, therefore these were combined and their means used to represent STH egg concentrations in sludge from Durban. *Ascaris* spp was the most abundant (722 (±534)/g), with *Toxocara* spp being the least (43(±57)/g) (Table 2). The difference between the total and viable STH egg concentrations was statistically significant for all the types of eggs detected except for *Taenia* spp and *Toxocara* spp. Table 2 presents the viable STH egg concentrations. Viable egg concentrations varied significantly over the duration of the study. As shown in Fig. 1, *Ascaris* spp and hookworm eggs showed the largest variation, with high concentrations for these STHs (*Ascaris* spp and hookworm) in January with decrease in concentrations from February to March. There was an increase again in *Ascaris* spp and hookworm egg concentrations from July to September in the case of *Ascaris* spp and August in the case of hookworm.

**Table 2:** Mean (±SD) concentration of STH eggs per gram of sludge from Durban, South Africa.

	Mean (±SD)total STH eggs/g		Mean (±SD)viable STH eggs/g	
	Durban	Senegal	Durban	Senegal
*Ascaris* spp	722 (±534)	1079(±114)	369(±260)	769(±107)
Hookworm	334(±246)	257(±72)	146(±154)	186(±172)
*Trichuris* spp	154(±148)	1647(±270)	49(±49)	84(±17)
*Taenia* spp	54(±62)	N/A	20(±27)	N/A
*Toxocara* spp	43(±57)	N/A	22(±32)	N/A

*Ascaris* spp, *Trichuris* spp and hookworm were the only STH egg detected in the sludge samples from Dakar, with *Trichuris* spp eggs the most abundant (1647(±270) eggs/g), the variation in egg concentration of these STHs was statistically significant (p value ≤ 0.05). Viable STH eggs were significantly lower (p value ≤ 0.05) than the total STH egg counts as expected. Table 2 presents the mean concentrations and variation of the STH eggs recorded.

STH egg concentrations in sludge from Dakar were significantly higher (p value ≤ 0.05) as compared to concentrations in Durban for STHs for *Ascaris* spp and *Trichuris* spp. However, the concentration of hookworm was higher in Durban (334(±246)/g) than in Dakar (257 (±72)/g).

### 3.2 Reduction viable STH egg concentration over a 90 day drying period

It was observed that mean concentrations of viable STH eggs reduced over the 60 day drying period (maximum days of sludge drying at the WWTPs), with mean viable STH egg concentrations of 826(±93) eggs/g in the fresh sludge. After 60 days of drying, concentration of viable STH eggs was 625(±30) eggs/g, however extension of the drying by approximately a month resulted in further reduction in viable STH eggs. After 90 days of drying, 406(±15) eggs/g were viable. A linear decay model was fitted to this data to derive a decay rate of 0.0056 per day. Table A1 in Appendix presents the results of the decay assessment.

### 3.3 Risk of *Ascaris* spp infection for farmers during the spread of sludge

Application of the sludge (after 60 days of drying) resulted in varying degrees of *Ascaris* spp infection risks, with a higher mean risk for farmers in Dakar (9.9 × 10^−1^ (±1.3 × 10^−5^)) compared to farmers in Durban due to a lower concentration of *Ascaris* spp eggs in the sludge (Table 3). Multiple exposures to the sludge during the application resulted in a much higher risk of *Ascaris* spp infection annually, as presented in Table 3, irrespective of the study site. Variation in the viable *Ascaris* spp egg concentrations over the duration of the study as shown in Fig. 1 did not significantly affect the estimated risks of infections, hence the mean risk was reported.

**Table 3:** Mean risk (±90% CI) if *Ascaris* spp infection for farmers spreading sludge on their farms.

	Probability of infection from one time exposure	Annual probability of infection
Durban	7.9 × 10^−1^ (±1.7 × 10^−2^)	1.0 (±1.1 × 10^4^)
Dakar	9.9 × 10^−1^ (±1.3 × 10^−5^)	1.0 (±3.5 × 10^−10^)

### 3.4 Risk of *Ascaris* spp infection farmers associated with ingestion of sludge amended soil

**Fig. 2:**
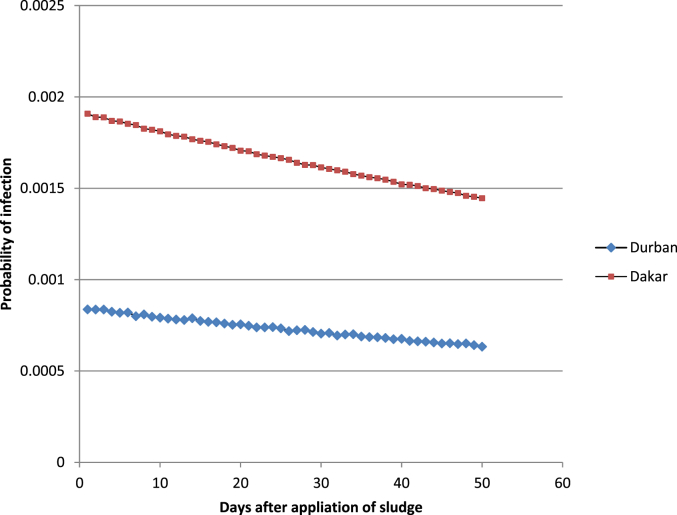
Median probability of infection with *Ascaris* spp from ingestion of sludge amended soil on different days after sludge application.

Ingestion of sludge amended soil will result in varying risks of infection depending on how many days after sludge application the exposure occurred. One day after sludge application leads to a median risks of 8.36 × 10^−4^(±1.13 × 10^−5^) for farmers in Durban and 1.91 × 10^−3^ (±1.09 × 10^−5^) in Dakar. Accounting for decay of the eggs the risks of infection reduced with time. Approximately one month (30 days) after application, risks were 7.14 × 10^−4^ (±9.61 × 10^−6^) in Durban and 1.62 × 10^−3^ (±9.27 × 10^−6^) in Dakar. Fig. 2 shows the median probability of infection due to ingestion of sludge amended soil depending on the day of exposure, with a maximum duration of 50 days after application.

### 3.5 Risk of *Ascaris* spp infection for consumers of lettuce grown on sludge amended soil

**Fig. 3:**
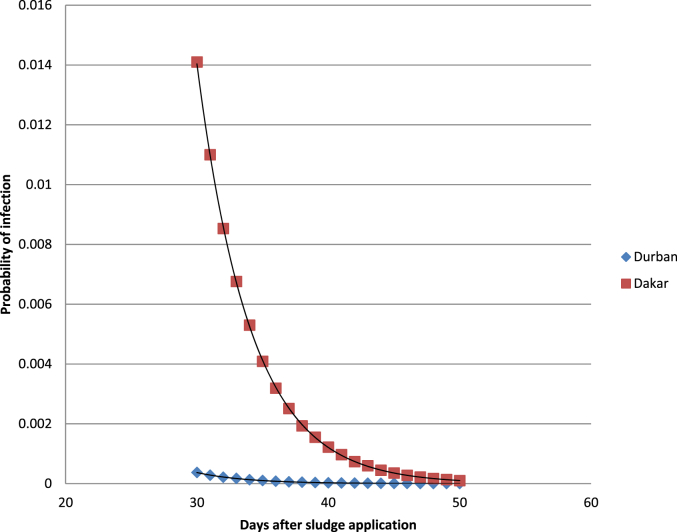
Median probability of infection with *Ascaris* spp from consumption of lettuce contaminated with sludge on different days after sludge application.

Harvest of lettuce after 30 days of sludge application resulted in risks estimates of 2.7 × 10^−4^ (±1.24 × 10^−4^) for consumers in Durban and 4.5 × 10^−3^ (±1.9 × 10^−4^) for consumers in Dakar and by the 50^th^ day the risks are expected to be reduce further to 1.21 × 10^−6^ (±1.18 × 10^−4^) in Durban and 6.24 × 10^−5^ (±1.91 × 10^−4^) for consumers in Dakar. Fig. 3 shows the probability of infection depending on the day of harvest, indicating that risks of ascariasis in Durban are very low as compared to Dakar.

## 4 Discussion

*Ascaris* spp, hookworm, *Trichuris* spp, *Taenia* spp and *Toxocara* spp were the common soil-transmitted helminth eggs in the sludge, except for Dakar, where *Taenia* spp and *Toxocara* spp were not detected. The difference in prevalence and concentration of these STH eggs is reflective of the prevalence of infections within the respective populations. STH eggs detected in sludge from Durban correspond to STH infections within this study area (Mkhize-Kwitshana and Mabaso, 2014, Molvik et al., 2017, Appleton et al., 2009). STH egg concentration in fresh faeces collected from urine-diverting latrines within the same province (KwaZulu Natal) contained from 1–1425 eggs of STH per gram (Trönnberg et al. (2010) which represent both a high concentration and a high variability of STH eggs in faecal matter from infected individuals. In Senegal, the most common STH infections are *Ascaris* spp, hookworm and *Trichuris* spp (Tine et al., 2013), similar to the STHs detected in this study. Globally, *Ascaris* spp, hookworm and *Trichuris* spp are the most common STH infections, accounting for approximately 1.5 billion infections mainly in sub-Saharan Africa, the Americas, China and East Asia (WHO, 2017). STH egg concentration in sludge from main wastewater treatment plants is mainly dependent on their concentration in raw wastewater, which in turn is dependent on infection prevalence in the population. STH eggs are particles which forms part of the total suspended solid component of wastewater and therefore the reduction and sedimentation of suspended solids in wastewater treatment processes result in their (STH eggs) removal as well (Jimenez-Cisneros, 2006). This results in an accumulation of STH eggs in the sludge. The concentration of STH eggs found in this study is similar to concentrations in Mexico (Pecson et al., 2007) and Ghana (Koné et al., 2007). In other studies lower concentrations have been found, e.g in France (Gantzer et al., 2001), Brazil (Bastos et al., 2013) and Morocco (Moubarrad and Assobhei, 2007).

With total viable STH eggs ranging from 25-185 per gram, the sludge from both study areas exceeded the USEPA standards and WHO guideline values or the South African regulations (which are an adaptation of the USEPA regulations). These high viable STH egg concentrations after approximately 2 months of drying could be attributed to the conditions under which the sludge was dried. The sludge was air dried without mixing for periods ranging from 60 to 90 days depending on demand from farmers and other users.

The ambient temperatures of 24 °C in Durban and 28 °C in Dakar (www.climate-data.org) and the duration of drying is thus inadequate to inactivate a greater proportion of the eggs. Composting at ambient temperatures of 20–30 °C for 1–4 months has been shown to have no effect on *Ascaris* spp egg viability, however elevated temperatures of 40–50 °C decreases egg viability within hours to two weeks (Berendes et al., 2015, McKinley and Parzen, 2012, Szabová et al., 2010).

Viscous heating at temperatures over 70 °C and 80 °C can achieve complete inactivation of *Ascaris* spp eggs within 15 and 5 s respectively (Belcher et al., 2015, Naidoo et al., 2017), additionally, UV irradiation of about 200 to 2000 J/m^2^ (using a UV lamp) results in the activation of between 0 and 1.5 log of *Ascaris suum* eggs (Brownell and Nelson, 2006).

With the assumption that sludge will be collected for land application after 60 days of drying in both study sites, the risks of ascariasis was found to be very high for farmers involved in the practice and above the WHO tolerable risks value (10^−4^) for sludge application in agriculture (WHO, 2006). The mean risks of infection for farmers in Durban (10^−4^) during the application stage was found to be similar to risks estimates from Ghana, however the estimates from Dakar were one magnitude higher (10^−3^) (Seidu et al., 2008b). Due to the labour-intensive nature of farming in many African countries including Senegal and parts of South Africa, ingestion of soil is a likely route of further risks but accounting for the decay of *Ascaris* spp eggs over time, the risks of infection will reduce.

Despite these anticipated reductions in viable *Ascaris* spp eggs, the annual risks of infections still exceed the WHO tolerable risks value in both study areas. Based on the decay of the viable eggs determined in this study further treatment is needed to reduce the risks of infections. The decay rates determined might be due to the ability of these eggs to survive for longer periods under favourable conditions (WHO, 2015). Additionally multiple application of sludge on the same piece of land may result in egg accumulation in soil (Seidu et al., 2008a), which may increase the doses ingested by the farmers. All these factors may result in higher risks of ascariasis than were estimated here.

Comparatively consumers of vegetables grown on sludge amended soils in Durban are less likely to get infected with ascariasis after 30 days of sludge application, compared to Senegal. In Senegal harvest of lettuce after 30 days of sludge application will however still result in risks of infections. To protect consumers in Senegal from *Ascaris* spp infections, it is suggested that harvesting be done at least 40 days after the sludge application and most preferably after further treatment. It must be noted that these risks estimates are based on the assumption of decay of the *Ascaris* spp eggs, however these eggs are fairly resistant and survive for longer periods of time. In addition, contamination of lettuce with soil might also result in higher contamination levels than have been estimated due to egg accumulation in soil.

To reduce the estimated risks of ascariasis there is the need for further treatment of the sludge to protect public health, composting using a pH elevation 12 (Gantzer et al., 2001), as well as thermophilic composting in a vessel (aerobic/anaerobic) (Haug, 1993, Eller et al., 1996), may all result in 3 log reductions in helminth egg concentration within 1–5 days. Low cost options such as composting in pH > 9 may also result in 3 log reductions but after a storage duration of 6 months (Chien, 2001). Windrow thermophilic composting for 3 months may also achieve 1.5–2 log reduction (Koné and Struass, 2004). Additionally, viscous heating may result in over 90% to complete inactivation of STH eggs providing an additional inactivation barrier before composting. These additional treatment barriers may reduce the viable STH egg concentrations to levels that will result in probability of infection within the WHO guideline value (10^−4^) translating into 10^−6^ Disability Adjusted Life Years, rendering the sludge safe for land application.

## 5 Conclusion

Soil-transmitted helminth egg concentration in sludge from both Durban and Dakar were found to be high, which is consistent with prevalence of infection within the study areas. These concentrations are also similar to results from other geographical locations with similar socio-economic status as the study areas. Although viable STH egg concentrations were significantly lower than the total STH egg concentrations, these were above the WHO guideline value as the USEPA limit for sludge intended for unrestricted agricultural application. Due to this high concentration of viable STH eggs, risks of *Ascaris* spp infection for farmers spreading the sludge was found to be higher than the WHO tolerable risk value. Additionally consumption of lettuce grown on sludge amended soil was also found to result in higher risks of infection especially in Dakar, however delay of harvest till 40 days after sludge application reduces the risks below the WHO tolerable risk value. Therefore sludge from both study sites needs further treatment, such as composting under elevated pH or heat treatment, to reduce the concentration of viable STH eggs and therefore protect public health.

## Figures and Tables

**Table A1:** Reduction of viable STH egg count over a 91 day drying period at different depths on sludge.

	Day1	Day 7	Day 14	Day 21	Day 28	Day 35	Day 42	Day 49	Day 56	Day 63	Day 70	Day 77	Day 84	Day 91
Top	598.16	570.12	569.23	468.59	412.23	303.01	308.33	309.34	302.15	300.18	299.48	298.2	301.24	289.45
Middle	658.46	639.78	640.23	590.12	610.12	598.79	568.79	565.78	490.12	482.45	440.59	438.79	419.12	420.12
Bottom	620.45	623.12	602.89	597.45	548.97	578.98	580.26	576.45	560.45	561.04	559.78	520.15	512.23	408.15

AVG	625.69	611.01	604.12	552.05	523.77	493.59	485.79	483.86	450.91	447.89	433.28	419.05	410.86	372.57
SD	30.49	36.38	35.52	72.37	101.32	165.35	153.79	151.23	133.54	133.82	130.30	112.28	105.74	72.24
